# Management of Acquired Factor VIII Inhibitors With NovoSeven and Obizur

**DOI:** 10.7759/cureus.19145

**Published:** 2021-10-30

**Authors:** Jennifer L Miatech, Deepti Kantamani, M. Patrick Stagg

**Affiliations:** 1 Internal Medicine Residency Program, Baton Rouge General, Baton Rouge, USA; 2 Internal Medicine, Baton Rouge General, Baton Rouge, USA

**Keywords:** coagulation inhibitor, surgical management of obstetrical hemorrhage, factor viii, factor viii inhibitor bypassing agents (feiba), acquired hemophilia a (aha)

## Abstract

Acquired hemophilia A (AHA) is a rare hemorrhagic disorder caused by the production of autoantibodies against coagulation factor VIII (FVIII). AHA is associated with significant morbidity and mortality primarily as a result of bleeding. Although many disorders are associated with the development of these inhibitors, up to 50% of cases remain idiopathic. The approach to therapy involves an initial strategy often to control acute bleeding episodes followed by definitive treatment to eradicate the inhibitor with immunosuppressive agents. We present the case of a 63-year-old Caucasian male hospitalized for severe Covid-19 who developed bleeding due to an acquired FVIII inhibitor that had never been treated definitively. Our case presentation focuses on in-hospital management of this patient's acute bleeding episodes with by-passing agents and recombinant porcine factor VIII.

## Introduction

Acquired factor VIII (FVIII) inhibitors are antibodies of different IgG subclasses directed against epitopes on coagulation factor VIII molecules. The disorder, also known as acquired hemophilia A (AHA), causes significant morbidity and mortality, with severe bleeding in up to 90% of cases. Alloantibodies to FVIII develop in approximately 20-40% of hemophilia patients who receive frequent FVIII replacement. In the non-hemophiliac population, this disorder is rare with an estimated incidence of 1 to 4 per million/year [1]. FVIII autoantibodies may be associated with the postpartum period, autoimmune diseases, malignancies, infections, or medications, such as penicillin and sulfa antibiotics. Although it may be associated with several underlying pathologies, up to 50% of reported cases remain idiopathic [1, 2].

AHA-associated bleeding can range from mild ecchymosis to life-threatening bleeding. By-passing agents and recombinant porcine FVIII are established treatment modalities to achieve hemostasis. Currently, no randomized studies are available comparing the efficacy between these agents, and decision-making is often dependent upon clinical judgment and product availability. We present the case of a 63-year-old male hospitalized for severe Covid-19 who developed bleeding because of an acquired FVIII inhibitor that had never been treated definitively. The patient achieved hemostatic control with recombinant porcine factor VIII (susoctocog alfa [e.g. Obizur]) after initial management with recombinant factor VIIa (e.g. NovoSeven) did not completely control his bleeding. This case highlights clinical management of AHA-related bleeding outside of an institution with the robust resources of a hemophilia center.

## Case presentation

A 63-year-old Caucasian male with a medical history of type 2 diabetes, temporary tracheostomy placement after West Nile infection in 2012, provoked deep vein thrombosis after debility from West Nile infection, and an idiopathic factor VIII inhibitor was admitted for severe Covid-19 viral pneumonia in January 2021. He was initially diagnosed with an acquired FVIII inhibitor in July 2016 after bruising and extremity swelling following minor arm trauma. Laboratory studies at that time revealed a white blood count of 12 K/uL with a normal differential, hemoglobin of 11.3 g/dL, and platelets of 333 K/uL. Coagulation studies revealed a prothrombin time (PT) of 13.9 sec, a partial thromboplastin time (PTT) of 62 sec, an international normalized ratio (INR) of 1.07, and a thrombin time of 15.4 seconds. A mixing study revealed the presence of inhibitors. Factor VIII activity level was <1%, with an inhibitor level of 200 Bethesda units. Lupus anticoagulant was negative. Factor IX, XI, XII, XIII, and von Willebrand factor activities were normal. Rheumatologic serologic testing was negative, as were Hepatitis B and C. The patient was first treated with steroids, but declined a plan to initiate eradication therapy with cyclophosphamide or rituximab due to cost.

His next presentation was in May 2018 with excessive bleeding and significant edema of the posterior compartment of his calf after a ground level fall. He received two units of packed red blood cells for a hemoglobin of 7.5 g/dL. His PTT was prolonged at 50 sec. His bleeding resolved with factor VIII inhibitor by-passing activity (FEIBA) 50 units/kg. Unfortunately, he never followed up to pursue eradication therapy.

During this admission in January 2021 for Covid-19, he developed worsening hypoxemia requiring mechanical ventilation and central venous catheter placement. His admission hemoglobin was 12.5 g/dL, platelets were 313 K/uL, and PTT was 81 sec with an INR of 1.1. Routine prophylactic anticoagulation was held on admission due to a prolonged PTT. His oxygenation continued to worsen, prompting concern for possible pulmonary embolism. Further evaluation with computed tomography angiography was deemed unsafe as the patient was clinically unstable for transfer to the radiology department. Therapeutic enoxaparin was provided empirically. Following administration of empiric anticoagulation, he developed excessive oozing from his central venous access site with subsequent hematoma formation. The hematology service was consulted and abruptly discontinued enoxaparin. He achieved adequate hemostasis without further intervention.

The patient then required a repeat central venous catheter placement two weeks later, for which he received a one-time dose of prophylactic and post-procedure recombinant human factor VIIa (NovoSeven) at 90 mcg/kg. FEIBA was not available at our institution. Seven days later, the patient required a tracheostomy and percutaneous endoscopic gastrostomy tube placement. He received two doses of NovoSeven at 90 mcg/kg perioperatively along with one unit of packed red blood cells with only minor bleeding. Post-operatively he developed continuous oozing from his tracheostomy site raising concerns for possible airway compromise, for which he received Novoseven every 2 hours. Recombinant porcine factor VIII (susoctocog alfa [e.g. Obizur]) administration was considered, unfortunately, was not available at our institution, and delivery was delayed due to severe weather conditions. He continued to have oozing despite NovoSeven. Obizur became available two days later and he received a dose of 9,000 units (100 units/kg) with rapid resolution of his bleeding. Ten days later his oozing from the tracheostomy site recurred, and two additional doses of 9000 units of Obizur were given 48 hours apart with successful cessation of bleeding. He did not have any further bleeding episodes and was transferred to a long-term acute care facility for mechanical ventilation weaning.

## Discussion

The development of acquired FVIII inhibitors, otherwise known as acquired hemophilia A, is rare. The most common clinical scenario for their development is in hemophilia patients who receive frequent FVIII replacement. The estimated incidence is 1 to 4 million/year in the non-hemophilic population. These autoantibodies produce significant morbidity in the form of bleeding in up to 90% of cases and a mortality rate of 8% to 22% [1,2]. FVIII autoantibodies are associated with the postpartum period, autoimmune disease, malignancy, infection, and medications, although 50% of cases are idiopathic. Autoantibody detection is typically biphasic with a small peak between 20-30 years due to postpartum inhibitors, with the more prominent peak in patients between 60-80 years [2]. The clinical presentation of patients with acquired hemophilia differs from those with hereditary hemophilia A as hemorrhage into the skin, soft tissues, muscle, or mucous membranes (such as gastrointestinal and urological) frequently occurs, whereas hemarthrosis is uncommon.

The diagnosis of AHA is often based on the initial detection of an isolated prolongation of activated partial thromboplastin time, which cannot be corrected by normal plasma (mixing study), and subsequent identification of a reduced FVIII level with evidence of FVIII inhibitor activity (titrated using the Bethesda assay or its Nijmegen modification). The treatment priorities are first to arrest any acute bleeding then eradicate the factor VIII autoantibody with immunosuppressive agents. Spontaneous resolution of inhibitors has been reported in up to a third of cases, however, this is more commonly observed in pregnancy or drug-related AHA. Prednisolone at 1 mg/kg/day results in inhibitor eradication in approximately 30% of patients, with the addition of cyclophosphamide increasing the response rate to 60-70% [1]. Other agents can be used to achieve eradication including azathioprine, vincristine, mycophenolate mofetil, and rituximab. Immune tolerance induction protocols, similar to the ones used for the treatment of alloantibodies in congenital hemophilia, have also been proposed for the eradication of FVIII autoantibodies. These protocols demonstrated inhibitor eradication in 90% of patients [3]. The choice of the most appropriate therapeutic strategy will depend on the size and severity of the bleeding and the inhibitor titer. Treatment options for bleeding include desmopressin (DDAVP), factor VIII concentrates, by-passing agents such as recombinant human factor VIIa (rFVIIa; e.g., NovoSeven) and activated prothrombin complex concentrates (aPCC; e.g., factor eight inhibitor by-passing activity [FEIBA]), and recombinant porcine sequence factor VIII concentrate (e.g., susoctocog alfa [Obizur]).

Non-life-threatening bleeding and low inhibitor titers (e.g. <5 Bethesda units) can occasionally be managed with DDAVP. Replacement with factor VIII concentrates is often unable to overcome the inhibitor burden completely. In patients with life-threatening bleeding or high-titers, two strategies are available to achieve hemostatic control of acute bleeding, including the use of by-passing agents or therapies to raise the level of circulating FVIII. In current clinical practice, by-passing agents are the most utilized first-line treatment with activated prothrombin complex concentrates or rFVIIa. FEIBA, currently the only approved aPCC in the United States for AHA, is a plasma-derived concentrate consisting of activated factor VII and non-activated factors II, IX, and X. RFVIIa contains only recombinant activated factor VII. Both agents are considered safe with similar efficacy of >80%, rFVIIa is often preferred among clinicians due to its viral safety [4-6]. FEIBA is a vapour-heated plasma-derived coagulation factor concentrate with the potential for viral transmission, although no cases of human immunodeficiency virus or hepatitis transmission have been reported in the literature. Concerns surrounding the possibility of trace amounts of FVIII in FEIBA producing an anamnestic response have not been definitively answered [7]. FEIBA and rFVIIa control bleeding by promoting thrombin generation in all plasmas except those deficient in Factor V [8]. These by-passing agents have been associated with thromboembolic complications, although rare and mostly seen after large and repeated doses exceeding the recommended doses [9]. The FENOC study, a randomized comparison of FEIBA and NovoSeven, found similar hemostatic efficacy between the products. It is important to note that patients who received both, approximately a third of patients demonstrated better efficacy with one product over the other [4]. Therefore, it is essential to individualize by-pass therapy for clinical management.

The limitations of by-passing agents are the lack of routine clinical monitoring and the risk of thrombotic complications, which has led to the development of alternative factor VIII concentrates. Porcine factor VIII (pFVIII) was studied and thought to be less inactivated by FVIII inhibitors. A porcine plasma-derived pFVIII (Hayate:C) was removed from the market in 2004 due to viral safety and hypersensitivity reaction concerns [10]. A safe recombinant porcine factor VIII product known as Obizur was designed to lack the B domain subsequently, decreasing FVIII antibody formation. Obizur achieves hemostasis through the binding of human von Willebrand factor and becomes activated by thrombin to achieve hemostasis. Unlike by-passing agents, Obizur replaces the missing coagulation protein and enables measurement of FVIII activity using available standard FVIII assays, thereby, guiding dosing and enhancing treatment efficacy and safety.

Clinical trial data evaluating the efficacy of Obizur reported 86% success of achieving hemostatic control [11]. The authors also found a higher rate of treatment success among subjects receiving Obizur as ‘first-line’ therapy compared to subjects treated with another hemostatic agent prior to treatment. No serious adverse events with Obizur were reported in this trial. A limitation to the study was the highly variable dosing. Fosbury et al. reported that the response to treatment is unique to each patient and may be influenced by the presence and development of anti-pFVIII antibodies.

Treatment delays can result in potential harm if clinically significant bleeding is not managed urgently. Current efficacy and safety data do not appear to favor by-passing agents or recombinant porcine FVIII. In clinical practice, front-line management is often determined by product availability and institutional laboratory testing. Monitoring of FVIII activity and quantifying rpVIII antibodies is possible with Obizur, although often not feasible at all institutions during acute management. We managed this patient clinically as laboratory measurements were not readily available and he did not demonstrate life-threatening bleeding. The patient developed minor bleeding episodes during his hospitalization that was initially managed successfully with rVIIa, and when bleeding continued he received Obizur without further bleeding or complications.

## Conclusions

This case highlights the management of AHA-related bleeding clinically at an institution without the robust resources of a hemophilia center. Individual institutional approaches will vary based upon product availability and laboratory capabilities. Treatment should be started with an agent that is immediately available. A clear consensus on the management of this disorder has not yet been established. Contacting a hemophilia center can be beneficial for successful management. It is possible to successfully manage acute AHA bleeding clinically. Further studies are needed to address the assessment and management of these patients.
